# A robust and verifiable federated learning framework for preventing data poisonous threats in e-health

**DOI:** 10.3389/fpubh.2026.1762346

**Published:** 2026-03-17

**Authors:** Etidal Alruwaili, Tarek Moulahi

**Affiliations:** Department of Information Technology, College of Computer, Qassim University, Buraidah, Saudi Arabia

**Keywords:** blockchain, federated learning, healthcare analytics, poisoning attacks, security, trustworthy AI

## Abstract

**Introduction:**

Federated Learning (FL) has become an attractive approach for e-health because it allows multiple institutions to collaboratively train machine learning models without directly sharing sensitive patient data. Despite these advantages, FL systems are still susceptible to poisoning attacks in which malicious participants manipulate model updates to degrade performance or embed hidden backdoors. Such threats raise serious concerns for medical applications, where reliability, transparency, and regulatory compliance are essential.

**Methods:**

In this work, we introduce FedSecure-Chain, a modular framework designed to improve the reliability of federated learning environments. The proposed approach combines three phases: an anomaly detection stage applied before aggregation to identify suspicious client updates, a robust aggregation strategy to limit the influence of potentially malicious contributions, and a lightweight blockchain layer that records model updates and client trust information to ensure traceability and auditing. The framework was evaluated on Breast Cancer datasets using TabNet and compact multilayer perceptron (MLP) models under several poisoning attack scenarios and different non-IID data distributions.

**Results:**

The experimental evaluation indicates that integrating anomaly detection with robust aggregation significantly reduces the impact of poisoning attacks on the global model. In addition, the blockchain logging layer enables transparent tracking of model updates while introducing only limited overhead. Overall, the proposed framework maintains stable model performance even in the presence of adversarial participants.

**Discussion:**

The results suggest that combining defensive learning strategies with transparent logging mechanisms can strengthen trust in federated healthcare systems. By improving resilience to adversarial manipulation while keeping computational and operational costs manageable, Our method represents a practical step toward secure and trustworthy federated learning for healthcare applications.

## 1 Introduction

The adoption of machine learning in healthcare has accelerated in recent years, supporting tasks such as early diagnosis, risk prediction, and personalized treatment planning. Yet clinical data sensitivity and strict regulations make centralized data collection difficult. Federated Learning (FL) addresses this challenge by enabling institutions to train shared models without exposing raw patient data (1–3). By keeping data local and transmitting only model updates, FL aligns with HIPAA and GDPR and leverages heterogeneous patient populations across hospitals and clinics (3). Many FL systems also incorporate differential privacy, secure multiparty computation, and homomorphic encryption to strengthen data protection (1, 3, 4). These developments have supported applications in medical imaging, electronic health record analysis, and genomic medicine.

Despite these benefits, real-world FL still faces challenges related to non-IID data, communication overhead, and model heterogeneity across institutions (2). More importantly, FL introduces new security risks because adversaries can poison shared model updates. Even a small proportion of malicious clients can substantially degrade accuracy and recall (5–7). In healthcare, label-flipping and backdoor attacks are especially dangerous because they may induce targeted misclassifications or hidden behaviors (8). Such threats are critical in safety-sensitive settings where model reliability directly affects clinical decisions (9, 10). Existing defenses, including robust aggregation and anomaly detection, remain limited, particularly when facing multiple attack types or when institutions require transparent and verifiable audit trails.

Realistic FL deployments in healthcare operate under inherently heterogeneous and non-IID data conditions, where each institution serves different patient populations and employs varied clinical practices. This natural variability is not merely a statistical challenge; it significantly amplifies security risks by making the global model more sensitive to adversarial perturbations and creating opportunities for sophisticated, multi-vector poisoning strategies (11, 12). Attackers can exploit this fragility by manipulating labels, perturbing input features, crafting clean-label samples, or embedding subtle backdoor triggers. Furthermore, compromised clients can directly inject malicious gradients into the training process. The combination of non-IID data and these multi-pronged attacks represents one of the most challenging threats for federated systems, as defenses effective against a single, isolated attack often fail when facing mixed or adaptive adversaries (11, 13).

This gap has motivated specialized defense frameworks. SecFLH applies cosine distance analysis and HDBSCAN clustering to isolate malicious updates in non-IID settings (14), while FLDA uses local data mixup and gradient detection to counter data poisoning under heterogeneity (13). Although these approaches advance the field, the interaction between data heterogeneity and adversarial robustness still creates vulnerabilities that require integrated solutions (11). FedSecure-Chain addresses this by combining spectral anomaly detection with a verifiable blockchain trust anchor for realistic healthcare FL environments.

Most existing defenses focus on isolated poisoning threats such as label flipping or specific model-poisoning attacks and often fail against stronger adversaries who coordinate multiple vectors or exploit non-IID clinical data (15). Recent work has begun to address these challenges, including FLTracer for cross-round provenance analysis (16), SecFLH for multi-step clustering in healthcare IoT (14), and FLAegis for robust aggregation against multiple attack types (17). However, a major gap remains in verifiable accountability. These advanced methods still function as black-box defenses without immutable audit trails, limiting adoption in healthcare environments governed by HIPAA and GDPR where updates, client behavior, and defense outcomes must be fully traceable. Current FL systems, even with strong detection capabilities, struggle to provide this level of transparency needed for clinical trust.

To address these limitations, we introduce FedSecure-Chain, a modular and practical framework designed to improve the robustness and trustworthiness of federated learning in healthcare analytics. The framework combines three complementary defense layers:

- Pre-aggregation anomaly detection, which estimates the similarity and consistency of client updates to identify suspicious or poisoned contributions before aggregation.
- Robust aggregation mechanisms, which limit the influence of adversarial clients even when the anomaly detector is uncertain or when multiple attack vectors are present.
- A lightweight blockchain layer, which provides immutable logging of model update hashes and client trust metrics, enabling transparent auditing and regulatory compliance with minimal overhead.

We evaluate FedSecure-Chain using deep learning backbones suited for tabular medical data, including TabNet and compact MLPs, on the Breast Cancer datasets. Our experiments include five distinct poisoning attacks and several non-IID data scenarios, offering a realistic assessment of the framework's resilience.

The remainder of this paper is organized as follows. Section 2 reviews related work on federated learning in healthcare, poisoning attacks, defensive techniques, and blockchain-based audit solutions. Section 3 presents the FedSecure-Chain framework, including its architecture, adversary model, anomaly detection module, robust aggregators, and blockchain protocol. Section 4 details the experimental setup, dataset, evaluation metrics, and ablation study design. Section 5 reports and analyzes the results under different attacks and system configurations. Finally, Section 6 concludes the paper and outlines potential directions for future research.

## 2 Related work

This section highlights the key themes that shape current research on federated learning in healthcare. We briefly outline how FL is being applied in medical settings, the vulnerabilities that make it susceptible to poisoning, the main strategies proposed to defend against such threats, and the emerging role of blockchain in strengthening transparency and security within FL ecosystems.

### 2.1 Federated learning in healthcare: benchmarks and applications

Federated learning has been widely explored in healthcare due to its ability to support collaborative model training without exposing sensitive patient data, thus maintaining compliance with regulatory frameworks such as HIPAA and GDPR (1, 18). Prior work highlights the benefits of FL across diverse medical applications including disease prediction, medical image analysis, and electronic health record (EHR) modeling, where distributed training enables models to leverage heterogeneous patient populations that cannot be centralized because of privacy and institutional constraints (1, 19). Studies consistently show that FL can achieve performance comparable to centralized approaches while preserving privacy and supporting multi-institution collaboration (18).

Several benchmark investigations further emphasize the practical challenges that arise when deploying FL in real healthcare environments. Non-IID data distributions, uneven local sample sizes, and hardware heterogeneity across institutions can hinder convergence and stability (20, 21). Communication overhead and unreliable network conditions add additional operational constraints, especially at scale. To mitigate privacy risks associated with gradient sharing, many systems adopt mechanisms such as differential privacy, secure multiparty computation, and homomorphic encryption, but these protections often come with trade-offs in accuracy or computational efficiency (1, 2, 21). Overall, while FL presents a compelling framework for privacy-preserving analytics, its deployment in healthcare continues to face significant challenges that must be addressed to ensure secure, reliable, and efficient operation across diverse clinical settings (1, 2, 20).

To highlight the landscape of existing research and clarify the gaps our work addresses, Table 1 presents key surveys and SoK studies on poisoning attacks and defenses in FL. Taken together, the comparative insights in this table underscore both the maturity and fragmentation of the FL security literature, motivating our subsequent discussion of poisoning vulnerabilities and defense mechanisms in healthcare settings.

**Table 1:** Surveys and systematization on FL security and poisoning (2023–2025).

**Year and venue**	**Paper**	**Scope**	**Attacks covered**	**Defense families**	**Reviewer-useful notes**
2025, Complex and intelligent systems	(43)	Taxonomy of FL threats and defenses; relationships, pros/cons, future directions	Backdoor, Byzantine/model poisoning, adversarial examples	Robust aggregation, anomaly detection; overview across non-IID settings	Clear taxonomy to frame multi-attack threat model; useful to justify attack portfolio
2022, ACM DL (survey)	(44)	Unified review of poisoning in centralized and federated learning; goals, mechanisms, countermeasures	Label-flip, targeted backdoor, feature poisoning across centralized/FL	Data sanitization, augmentation, robust aggregation, certified defenses	Connects classical poisoning research to federated security extensions
2023, MDPI electronics	(45)	End-to-end overview of FL architecture, attack surfaces, and defense maps	Data poisoning (incl. label-flip), model poisoning, backdoor triggers	Client screening, clustering/outlier filtering, Krum/Trimmed-Mean	Contains figures illustrating FL workflow and attack taxonomy; good for introductory visuals
2025, arXiv (SoK)	(46)	Systematization of poisoning knowledge; benchmarking guidance; widely used FL algorithms	Mixed poisoning scenarios across FL algorithms (e.g., FedAvg variants)	Benchmarking protocols, standardized evaluation procedures	Supports standardized metrics and ablation reporting for your framework
2023, Information fusion	(47)	Extensive review and experimental study of FL threats and defenses	Comprehensive multi-attack coverage across FL	Comparative analysis of defense efficacy	Strong anchor survey for defining breadth and expected experimental rigor
2023, Survey (semantic scholar index)	(48)	Overview of poisoning attacks and defense strategies tailored to FL	Data/model poisoning classes summarized for FL	Defense strategy landscape (pre-/post-aggregation)	Secondary survey to triangulate taxonomy and tighten coverage

### 2.2 Poisoning attacks in FL

Poisoning attacks in FL have become an active area of research, as adversaries can influence the global model by manipulating local updates rather than raw data. Two major categories are commonly studied:

1. **Data-poisoning attacks**, such as label flipping, clean-label manipulation, feature injection, and targeted sample crafting, and
2. **Model-poisoning attacks**, which directly alter gradients or force malicious directions during local training.

Recent research demonstrates significant vulnerabilities in federated learning systems, where even small fractions of malicious clients can severely compromise model integrity. Data poisoning attacks can cause substantial drops in classification accuracy and recall with minimal malicious participation (5). These vulnerabilities are particularly pronounced under non-IID data distributions, where natural client heterogeneity makes malicious behavior harder to detect (22). Advanced collaborative backdoor attacks like CollaPois exploit this heterogeneity by distributing Trojan-infected models among compromised clients, enabling stealthy operation that bypasses existing defenses while maintaining normal performance on legitimate data (23). The threat landscape encompasses both data poisoning and model poisoning attacks, with malicious participants able to introduce backdoored functionality that activates only under specific triggers (24). Research indicates that the number of malicious clients has greater impact than poison concentration levels, and federated learning algorithms show varying resilience, with FedNova demonstrating superior robustness compared to FedAvg under adversarial conditions (22).

Table 2 highlights the diversity of poisoning attacks studied in FL and the defense mechanisms proposed. It demonstrates how different research efforts have quantified attack impact and proposed mitigation strategies across healthcare and general FL applications.

**Table 2:** Representative studies of poisoning attacks in FL.

**Paper**	**Attack type**	**Key findings**	**Defense proposed**
Shejwalker and Houmansader (49)	Model poisoning	1.5 × to 60 × higher accuracy reduction vs. previous attacks	Divide-and-conquer (DnC) defense
Shejwalker et al. (12)	Data & model poisoning	FL more robust in practice than previously thought	Simple, low-cost defenses effective
Tolpegin et al. (5)	Data poisoning (targeted)	Small percentage of malicious participants cause substantial accuracy drops	Malicious participant identification strategy
Nguyen et al. (50)	Backdoor attacks	Gradual poisoning via compromised IoT devices	Mitigation approaches discussed
Xia et al. (48)	Survey of poisoning attacks	Comprehensive classification of attack methods and targets	Three categories of defense strategies
Nowroozi et al. (51)	Label flipping & feature poisoning	Feature poisoning more effective than label flipping	Detection difficulty analysis
Zhang et al. (52)	Model poisoning	Model update inconsistency detection	FLDetector for malicious client detection
Ma et al. (53)	Encrypted model poisoning	30%–80% accuracy improvement in defense	ShieldFL using homomorphic encryption

Table 3 provides a generalized mapping of attack types in healthcare FL, describing the attack vectors, typical defenses, and considerations for experimental evaluation. Together with Table 2, these tables give a comprehensive view of both specific research outcomes and broader methodological guidance for mitigating poisoning attacks in Federated Learning.

**Table 3:** Attack–defense mapping for healthcare federated learning.

**Attack type**	**Typical FL vector**	**Representative sources**	**Common defenses**	**Notes for evaluation design**
Label-flipping (data poisoning)	Malicious clients invert or relabel targeted class locally before upload (44, 45)	MDPI Electronics survey, ACM poisoning survey (44, 45)	Trimmed-Mean, Median, Krum; CV-based filters and screening (45)	Include success-rate metrics, simulate 10%/20% malicious clients per round (45)
Backdoor / trigger-based	Insert rare triggers associated with target labels, persists through aggregation (43, 45)	FL security survey, FL attacks/defenses survey (43, 45)	Clustering/outlier filtering, robust aggregation, certified removal (44, 45)	Measure attack success under non-IID label/feature skew (43)
Feature manipulation (clean-label poisoning)	Shift/scale predictive features to degrade utility without overt label change (44)	Poisoning survey across centralized/FL (44)	Data sanitization/augmentation, anomaly detection pre-aggregation (44)	Report impact on AUROC + calibration to show clinical relevance (44)
Gradient poisoning / byzantine	Malicious updates (scaled/sign-flipped) disrupt global descent (43)	FL threats include Byzantine model-poisoning (43)	Byzantine-robust aggregation (Krum, Median), pre-agg outlier screening (45)	Track detection precision/recall on poisoned gradients (45)

### 2.3 Defenses: robust aggregation and anomaly detection

To counter poisoning attempts, a number of defensive strategies have been proposed. Recent research has addressed the limitations of static defensive strategies in federated learning through adaptive and multi-layered approaches. Haque et al. (25) introduced FedStrategist, a meta-learning framework that dynamically selects optimal aggregation rules using a contextual bandit agent, demonstrating that no single static defense is universally effective across diverse attack scenarios. Moyeen et al. (26) proposed FedChallenger, implementing a dual-layer defense combining zero-trust challenge-response mechanisms with a variant of Trimmed-Mean aggregation using cosine similarity and Median Absolute Deviation, achieving 3-10% accuracy improvements over existing methods. Chen et al. (27) developed FLRAM, which employs isolation forest and density-based clustering to detect gradient anomalies, followed by credibility matrix construction for trustworthiness evaluation. Erbil and Gursoy (28) focused on targeted attacks by proposing selective parameter extraction and embedding into low-dimensional latent space, combined with X-Means clustering to separate malicious from benign updates, achieving up to 95% true positive rates in malicious update identification.

A growing body of research has explored defensive strategies capable of mitigating poisoning risks in federated learning. These approaches range from robust aggregation rules and anomaly detection mechanisms to encrypted similarity checks and broader systematizations of defense families. Table 4 summarizes several influential works and highlights their contributions, performance characteristics, and the limitations they address. This overview helps contextualize our framework within the broader defense landscape and motivates the need for modular, multi-layered solutions such as FedSecure-Chain.

**Table 4:** Summary of key defensive strategies in federated learning.

**Paper**	**Year**	**Defense type**	**Specific method**	**Key performance**	**Limitations addressed**
Shejwalker and Houmansadr	2021	Robust Aggregation	Divide-and-Conquer (DnC)	2.5 × to 12 × more resilient than prior Byzantine-robust approaches	Weaknesses of existing Byzantine-resilient algorithms
Shejwalker et al.	2021	Simple Defense Mechanisms	Low-cost defenses for FL	Demonstrates that FL can be highly robust in practice with simple mechanisms	Challenges misconceptions about FL fragility in production
Zhang et al.	2022	Anomaly detection	FLDetector: model-update consistency checking	Accurately detects malicious clients across diverse attack types	Effective even under large numbers of malicious clients
Ma et al.	2022	Similarity-based detection	ShieldFL: secure cosine similarity with homomorphic encryption	30%–80% improvement in attack-defense accuracy	Enables privacy-preserving detection over encrypted updates
Xia et al.	2023	Survey / classification	Systematic taxonomy of FL defenses (three categories)	Comprehensive analysis of advantages and limitations	Clear structuring of defense strategies across FL settings

### 2.4 Blockchain for federated learning auditing and security

Blockchain has increasingly been explored as a complementary technology to strengthen security and accountability in federated learning. Its immutability, decentralized trust model, and consensus mechanisms offer a valuable foundation for defending against unreliable or malicious model updates. Prior work demonstrates this potential from several perspectives. Short et al. (29) introduced one of the earliest blockchain-enhanced defenses for model poisoning, showing that blockchain-based verification of client updates can meaningfully improve protection without requiring access to raw training data. Attiaoui et al. (30) expanded on this idea through a decentralized validation framework built on Proof-of-Stake, achieving resilience even when up to half of the participating clients were adversarial. Complementing these system-level defenses, Qammar et al. (31) conducted a systematic review revealing how blockchain can mitigate core FL challenges such as privacy exposure, unreliable parameter uploads, and high communication costs by distributing validation responsibilities across the network. Earlier work by Awan et al. (32) also leveraged blockchain's provenance tracking and tamper-proof storage to secure model update histories, addressing weaknesses in traditional semi-honest aggregation assumptions.

More recent studies highlight additional benefits and emerging gaps. Dong et al. (33) demonstrated that peer-to-peer blockchain voting can enhance trust management in federated settings, while Yu et al. (34) emphasized blockchain's usefulness in eliminating single points of failure common in centralized FL servers. At the same time, several limitations persist. Ning et al. (35) reported that blockchain-based FL often incurs significant communication and storage overhead, and most existing solutions concentrate on authentication and logging rather than offering strong resistance to poisoning attacks. Collectively, this body of work indicates that although blockchain provides a promising security substrate for FL, current approaches remain incomplete, and there is considerable opportunity for frameworks that integrate blockchain with stronger adversarial resilience and practical auditability.

Table 5 summarizes recent blockchain-enabled federated learning (BCFL) contributions in healthcare from 2023 to 2025, highlighting key system designs, privacy mechanisms, and reported performance claims.

**Table 5:** Blockchain-enabled federated learning for healthcare (2023–2025).

**Year and venue**	**Paper**	**Healthcare setting**	**On-chain functions**	**Privacy/ consensus**	**Reported results/claims**
2025, Scientific Reports	*Blockchain-enabled federated learning with edge analytics for secure and efficient EHRs (EPP-BCFL) (54)*	EHR management concept; CIFAR-10 evaluation	Lightweight logging and verification; PoS+BFT auditability	SMPC+ Differential Privacy; PoS+BFT consensus	95.2% accuracy, -43% latency reduction; robust to poisoning; DP ε = 1.0 gives 90.3% accuracy
2023, JMIR	*Architectural Design of a Blockchain-Enabled Federated Learning Platform for Algorithmic Fairness in Predictive Health Care (55)*	Simulated multi-center healthcare (5 institutions)	Aplos smart contract; Rahasak blockchain; fairness-aware logging	Decentralized FL + blockchain; microservices + Cassandra storage	Demonstrates fairness gains via FL + blockchain integration
2025, Scientific Reports	*Explainable federated blockchain framework with privacy-preserving AI optimization (PPFBXAIO) (56)*	Heart Disease (Kaggle) and Wisconsin Breast Cancer	SHA-256 update-hash logging; SHAP metadata on-chain for explainability	Privacy-preserving FL; optimization using LGOA inside BCFL system	95.07% accuracy / 95.98% F1; +109 TPS throughput; −81 ms latency vs baselines
2024, BMC Medical Imaging	*Toward blockchain-based federated learning in categorizing AIoMT devices (57)*	AIoMT device categorization in medical imaging	Decentralized processing; blockchain coordination	Permissioned blockchain for FL tasks	Methodology for decentralized FL categorization in IoT-healthcare
2025, ScienceDirect	*A Federated Learning Approach Toward Hybrid ... (Healthcare Collaboration) (58)*	Multi-entity healthcare collaboration	Hybrid on-chain coordination; transparent governance	Privacy-preserving hybrid FL	Secure and transparent collaborative FL across healthcare entities
2025, MDPI	*Smart and Secure Healthcare with Digital Twins: A Deep ... (59)*	Digital twins + healthcare FL	Ledger-based provenance for FL-twin interaction	Permissioned blockchain for healthcare twins	Architectural integration enabling secure, smart healthcare
2023–2025 Overview	*Blockchain Meets Federated Learning in Healthcare (Overview) (60)*	General BCFL improvements for healthcare	Edge nodes maintain blockchain; avoid single-point failure	Permissioned BCFL networks for reliability and auditability	Positions BCFL as enhancing privacy, security, and efficiency

## 3 The FedSecure-Chain framework

The FedSecure-Chain framework is a novel, integrated system designed to fortify FL against a wide spectrum of poisoning attacks and to provide a verifiable, tamper-proof audit trail for the entire learning process. By synergistically combining advanced anomaly detection, robust aggregation, and blockchain technology, FedSecure-Chain moves beyond traditional FL security models to offer a resilient and trustworthy decentralized learning environment.

### 3.1 System architecture: a three-layer defense model

Before diving into the technical details, it's worth understanding why this multi-layered approach matters. In healthcare federated learning, we're dealing with a perfect storm of challenges: sensitive patient data distributed across multiple hospitals, the constant threat of malicious actors trying to corrupt the learning process, and the critical need for transparency and accountability in medical AI systems. A single defense mechanism, no matter how sophisticated, simply isn't enough. Each layer in FedSecure-Chain addresses different attack vectors at different stages of the learning process, creating what security experts call “defense in depth.”

#### 3.1.1 Layer A: pre-aggregation anomaly detection—The first line of defense

Imagine a vigilant gatekeeper standing at the entrance of a fortress, carefully inspecting everyone who seeks entry. This is essentially what Layer A does for our federated learning system. Operating on the server side, this layer implements sophisticated screening mechanisms that examine client updates before they're allowed to influence the global model. Server-side screening of client updates using statistical-distance and spectral/graph criteria to down-weight or exclude suspected poisoned updates before any aggregation. This mitigates poisoning without committing suspicious updates to the global state and is especially important under non-IID client distributions that confound naive detectors (36, 37). The pre-aggregation detection system employs two complementary analytical approaches:

- **Statistical Distance Measures:** These techniques calculate how far each client's update deviates from expected patterns. It's similar to how a bank's fraud detection system flags transactions that don't match your usual spending behavior. If a hospital client suddenly submits an update that looks radically different from its historical patterns or from other similar clients, the system takes notice.
- **Spectral and Graph-Based Criteria:** These more advanced methods look at the mathematical structure of the updates themselves. By analyzing the eigenvalues and relationships between different parameters, the system can detect subtle patterns that might indicate a poisoning attempt–patterns that simple statistical measures might miss.

The beauty of Layer A is its proactive nature. By catching suspicious updates before aggregation, we prevent poisoned data from ever contaminating the global model. This is particularly crucial in healthcare settings where data distributions are often non-IID (non-Independent and Identically Distributed)–meaning different hospitals naturally have different patient populations and disease prevalences. In such environments, naive detection methods often cry wolf, flagging legitimate variations as attacks. Layer A's sophisticated algorithms, drawing on research by Singh et al. (36) and Liu et al. (37), are specifically designed to distinguish between natural variations in healthcare data and genuine malicious attempts. When the system identifies a suspicious update, it can down-weight its contribution or exclude it entirely, acting as a surgical filter rather than a blunt instrument.

#### 3.1.2 Layer B: robust aggregation—fortifying the core process

Once updates pass through the initial screening, they enter Layer B, where the actual aggregation happens. But this isn't just any aggregation, it's a Byzantine-robust process designed to withstand adversarial behavior even if some malicious updates slip through Layer A's net. Layer B employs multiple aggregation strategies, each with its own strengths:

- **Byzantine-Robust Methods:** These include techniques like Median aggregation (which takes the median value across all updates for each parameter), Trimmed-Mean (which excludes extreme values before averaging), and Krum (which identifies and aggregates updates that are most similar to each other). These methods are battle-tested approaches from the federated learning security literature, providing resilience against adversarial or statistically unusual update distributions.
- **FedAvg for Comparison:** The system also maintains the standard Federated Averaging (FedAvg) approach as a baseline. This allows researchers and practitioners to quantify exactly how much protection the robust methods provide compared to traditional approaches essentially measuring the cost of security in terms of model performance.

Think of these aggregation rules as a democratic voting system with built-in protections against manipulation. Even if a few malicious actors try to skew the results, the robust aggregation methods ensure that their influence is mathematically limited. The median approach, for instance, is inherently resistant to outliers, a few extreme votes can't shift the outcome. This is particularly valuable in healthcare federated learning, where the stakes of model corruption could directly impact patient care decisions.

#### 3.1.3 Layer C: lightweight blockchain verification—creating an immutable record

The third layer adds something that the first two cannot: verifiable provenance and tamper-evident audit trails. This is where blockchain technology enters the picture, not as a buzzword, but as a purposeful tool for creating trust and accountability. For each training round, Layer C creates an append-only record on the blockchain containing:

- **Update Hashes:** Cryptographic fingerprints of each client's contribution, allowing anyone to verify that updates weren't altered after submission.
- **Trust Scores:** Quantitative measures of each update's reliability based on Layers A and B's analysis.
- **Inconsistency Flags:** Automatic alerts when the system detects unusual patterns or potential security concerns.

Here's where FedSecure-Chain diverges from many blockchain implementations that buckle under their own weight. Drawing on recent work by Guo et al. (38) and Nezhadsistani et al. (39), this system employs a minimalist blockchain design. Rather than storing massive amounts of data on-chain, it records only essential metadata and hashes. The actual model updates remain off-chain, keeping the blockchain lightweight and efficient.

In healthcare AI, trust isn't optional, it's essential. When a hospital administrator or regulatory body asks, “How do we know this model wasn't tampered with?” or “Can you prove which hospitals contributed to this model?”, Layer C provides concrete answers. The immutable audit trail means that:

- Every contribution is traceable.
- No one can retroactively alter the training history.
- Suspicious patterns are automatically flagged and permanently recorded.
- Compliance with healthcare regulations (like HIPAA or GDPR) becomes demonstrably verifiable.

The real power of FedSecure-Chain emerges from the synergy between these three layers. Let's walk through a typical training round:

1. Submission: Multiple hospitals submit their locally-trained model updates to the central server.
2. Layer A Screening: The pre-aggregation detector analyzes each update, calculating statistical distances and examining spectral properties. Suspicious updates are flagged and down-weighted.
3. Layer B Aggregation: The surviving updates are aggregated using Byzantine-robust methods, ensuring that even if some malicious updates made it through screening, their impact is mathematically bounded.
4. Layer C Recording: The entire process, who submitted what, what trust scores were assigned, which updates were flagged, is cryptographically recorded on the blockchain, creating a permanent, verifiable record.

This orchestrated approach means that an attacker would need to simultaneously bypass statistical detection, overwhelm robust aggregation, and somehow alter blockchain records, a practically insurmountable challenge.

FedSecure-Chain's three-layer architecture represents a mature, thoughtful approach to securing federated learning in healthcare. It doesn't rely on a single silver bullet, but instead combines detection, mitigation, and verification into a cohesive defense strategy. Each layer compensates for the others' limitations: Layer A provides proactive filtering, Layer B ensures robust aggregation even when filtering isn't perfect, and Layer C adds the transparency and accountability that healthcare applications demand. For researchers and practitioners in medical AI, this architecture offers both strong security guarantees and practical feasibility.

**Figure 1:**
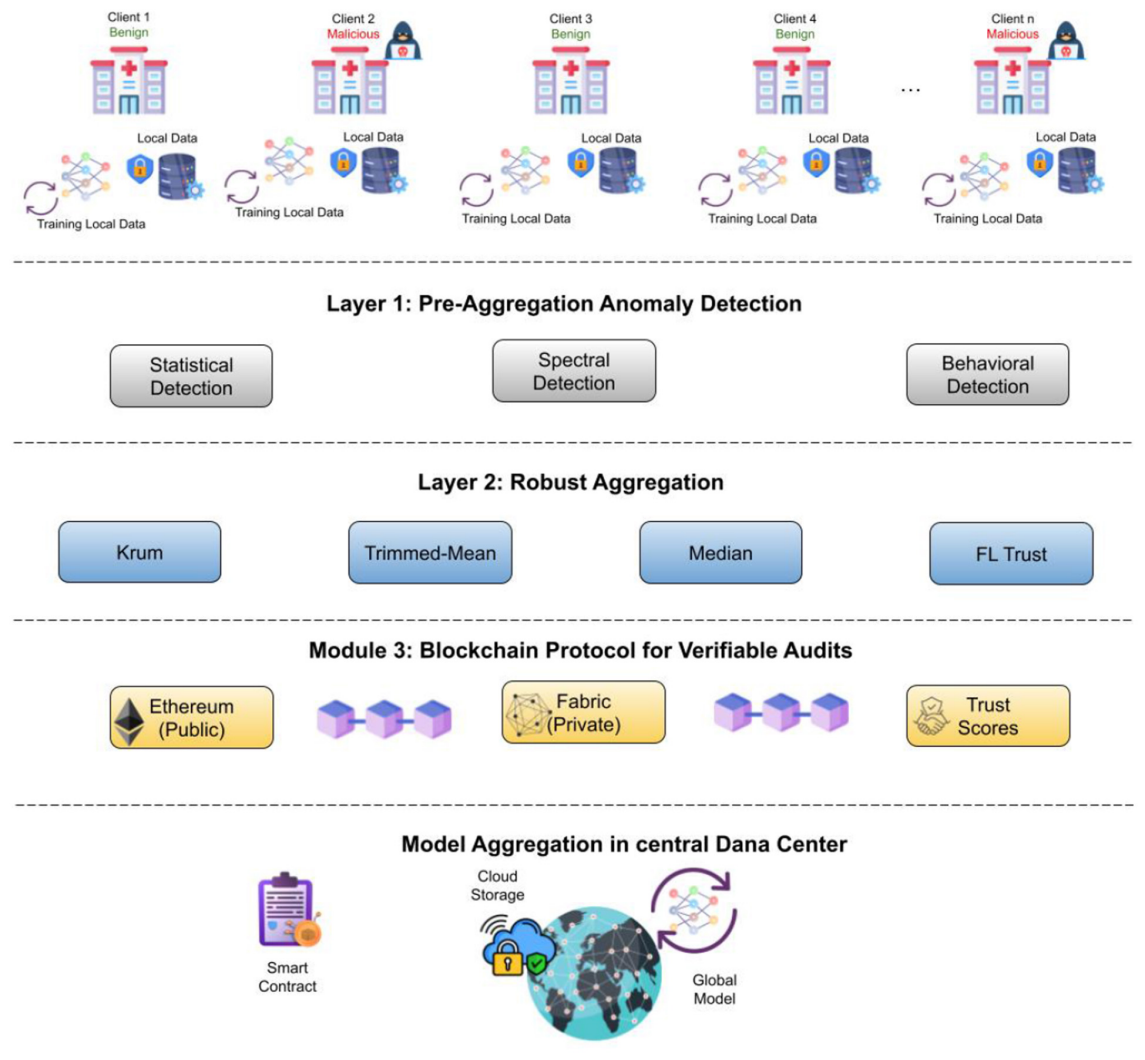
FedSecure-Chain: three layer defense architecture.

Figure 1 illustrates the three-layer architecture of FedSecure-Chain, showing the sequential flow from client update submission through pre-aggregation screening, robust aggregation, and blockchain verification.

### 3.2 Threat model and adversary specification

In federated learning for healthcare, participants may exhibit malicious behavior through compromised systems or deliberate manipulation. We consider a realistic adversarial setting where a fraction of clients attempts to corrupt the global model while evading detection. This threat model addresses practical concerns in medical federated learning, where data integrity directly impacts patient safety.

Our adversary has two primary objectives: first, to degrade the global model's performance on unseen data; second, to remain undetected by defense mechanisms. We assume the adversary understands the federated learning protocol and aggregation mechanism but cannot access other clients' data or observe real-time defense responses. This models realistic attackers who understand system architecture but lack omniscient capabilities.

#### 3.2.1 Poisoning attack configuration and parameter settings

To ensure full reproducibility and eliminate ambiguity in the adversarial evaluation, we explicitly specify all poisoning attack configurations and parameter values used in our experiments. This subsection summarizes the concrete settings adopted for each attack type, including the fraction of malicious clients, targeted classes, poisoning intensities, and whether attacks are static or adaptive across training rounds. These configurations are fixed prior to training and applied consistently across all experimental runs unless stated otherwise. Table 6 provides a comprehensive overview of the parameterization used for data poisoning and model poisoning attacks throughout the evaluation. the category is either DP (Data Poisoning) or MP (Model Poisoning).

**Table 6:** Poisoning attack configuration and parameter settings.

**Attack**	**Type**	**Mal. (%)**	**Target**	**Poison Params**	**Mode**
Label-flip	DP	10 / 20 / 30	Benign → Malignant	α = 0.3	Static
Clean-label	DP	10 / 20 / 30	Malignant	ϵ = 0.05	Static
Backdoor	DP	10 / 20 / 30	Malignant	Trigger = 5%	Adaptive
Byzantine (sign)	MP	10 / 20 / 30	All	λ = 5	Static
Byzantine (scale+noise)	MP	10 / 20 / 30	All	λ = 3, σ^2^ = 0.01	Adaptive

#### 3.2.2 Data poisoning attacks

Data poisoning corrupts the training process at the source by manipulating local datasets. These attacks produce seemingly legitimate model updates derived from genuinely trained models, making detection particularly challenging.

##### 3.2.2.1 Label-flip attacks

The adversary systematically flips training labels with probability ρ. For client *i* with dataset $D_{i}={\left\{\left(x_{j},y_{j}\right)\right\}}_{j=1}^{n_{i}}$, the attack produces:


$${\tilde{y}}_{j}=\{\begin{array}{ll} 1-y_{j} & \text{with probability }\rho \\ y_{j} & \text{otherwise} \end{array}$$ (1)

This directly undermines classification accuracy, with impact proportional to both ρ and the fraction of malicious clients.

##### 3.2.2.2 Clean-label poisoning

A sophisticated variant maintains correct labels while introducing imperceptible perturbations δ_*j*_ bounded by ||δ_*j*_||_2_ ≤ ϵ:


$$\begin{array}{l} {\tilde{x}}_{j}=x_{j}+\delta_{j} \end{array}$$ (2)

This attack is particularly insidious because labels remain valid, making poisoned data appear legitimate during inspection.

##### 3.2.2.3 Feature manipulation and backdoor injection

The adversary introduces systematic bias or backdoor triggers by manipulating feature distributions. For backdoor attacks, the malicious client optimizes a dual objective that balances maintaining model utility while embedding the backdoor:


$$\begin{array}{l} L_{\text{poison}}=\left(1-\beta \right)L_{\text{task}}\left(\theta ;D_{i}\right)+\beta L_{\text{backdoor}}\left(\theta ;D_{\text{trigger}}\right) \end{array}$$ (3)

where β∈[0, 1] controls the trade-off between task performance and backdoor effectiveness.

#### 3.2.3 Model poisoning attacks

Model poisoning directly manipulates gradient or parameter updates, bypassing the training process entirely. We focus on Byzantine gradient attacks, which represent the most general form of model poisoning.

For a benign gradient *g*_*i*_, Byzantine clients may submit:


$${\tilde{g}}_{i}=\{\begin{array}{ll} -\text{λ}g_{i} & \left(\text{sign-flip}\right)\\ \text{λ}g_{i} & \left(\text{magnitudescaling}\right)\\ g_{i}+\;\xi ,\;\xi \sim N(0,\sigma^{2}I) & \left(\text{additivenoise}\right) \end{array}$$ (4)

where λ>1 controls attack intensity. The sign-flip variant is particularly effective as it directly opposes gradient descent.

#### 3.2.4 Adversary capabilities and constraints

We model adversaries controlling a fraction α∈{0.1, 0.2, 0.3} of clients, representing 10-20% compromise rates. These proportions align with Byzantine fault tolerance assumptions (*f*<*n*/3 for *n* participants) and reflect realistic security scenarios.

**Adversary Knowledge:** The attacker knows the FL protocol, model architecture, and their local dataset distribution.

**Adversary Limitations:** The attacker cannot access other clients' data, observe aggregation in real-time, or know defense configurations in advance.

### 3.3 Module 1: pre-aggregation anomaly detection

Our first defense layer identifies malicious updates before aggregation. This pre-filtering prevents poisoned contributions from influencing the global model entirely, rather than merely diluting their impact. We employ complementary statistical and spectral methods for robust multi-perspective detection.

#### 3.3.1 Statistical distance-based detectors

Statistical detectors quantify how much each client's update deviates from the consensus. We implement four complementary distance metrics.

##### 3.3.1.1 Cosine similarity

For client *i* with flattened update vector *w*_*i*_, we compute similarity to the mean update $\bar{w}=\frac{1}{m}\overset{m}{\underset{j=1}{\sum }}w_{j}$:


$$\begin{array}{l} s_{i}=\frac{w_{i}^{⊤}\bar{w}}{||w_{i}|{|}_{2}||\bar{w}|{|}_{2}} \end{array}$$ (5)

We flag clients with *s*_*i*_ < μ_*s*_−τσ_*s*_, where μ_*s*_ and σ_*s*_ are the mean and standard deviation of similarities, and τ = 2 provides a two-standard-deviation threshold.

##### 3.3.1.2 Euclidean distance, Mahalanobis distance, and Z-score

We similarly compute Euclidean distance to the median update, Mahalanobis distance accounting for covariance structure, and per-dimension z-scores. Each provides a different geometric perspective on anomaly detection, with detailed formulations following standard statistical practice (40).

#### 3.3.2 Spectral and graph-based detectors

Spectral methods leverage graph theory to detect coordinated attacks. We construct a similarity graph where nodes represent client updates and edges encode pairwise similarity:


$$\begin{array}{l} S_{ij}=\exp\left(-\frac{||w_{i}-w_{j}|{|}_{2}^{2}}{2\sigma^{2}}\right) \end{array}$$ (6)

The graph Laplacian *L* = *D*−*S*, where *D* is the degree matrix, enables spectral analysis. We perform eigendecomposition and use eigenvector projections to identify outliers. Clients with high projections on large-eigenvalue eigenvectors exhibit structural deviation from the main cluster, indicating potential malicious behavior. The theoretical foundation follows spectral clustering literature (41).

##### 3.3.2.1 Multi-detector fusion

We combine detectors using majority voting: a client is flagged only if at least κ out of |D| detectors identify it as anomalous. This reduces false positives while maintaining high detection recall.

### 3.4 Module 2: robust aggregation layer

Robust aggregation mechanisms limit adversarial influence even when detection is imperfect. These aggregators provide Byzantine resilience by design, ensuring bounded impact from undetected malicious updates.

Algorithm 1 presents our complete defense pipeline, integrating detection, aggregation, and blockchain logging into a cohesive framework.

Algorithm 1 **FedSecure-Chain: integrated defense pipeline.**

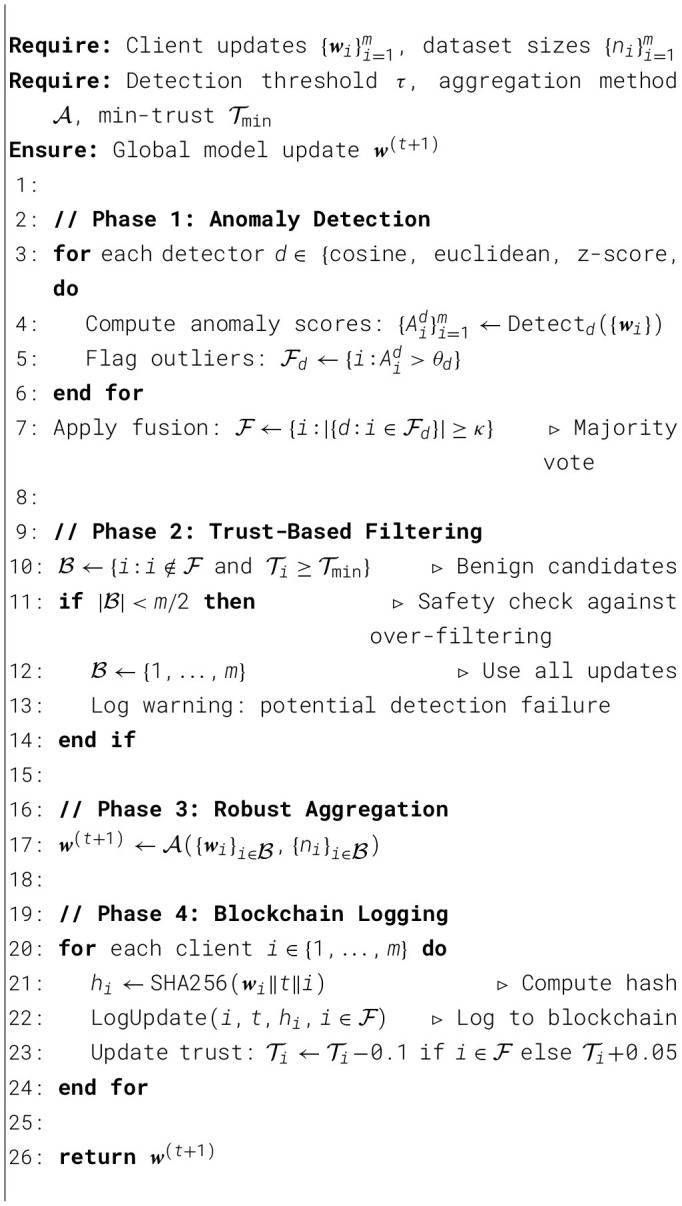

#### 3.4.1 Aggregation methods

We implement five aggregators with varying robustness-efficiency trade-offs:

*FedAvg* (Baseline): Weighted average by dataset size, $w^{\left(t+1\right)}=\underset{i\in B}{\sum }\left(n_{i}/N\right)w_{i}$ where $N=\underset{i\in B}{\sum }n_{i}$.

*Krum* (42): Selects the update closest to the majority. For each client, compute the sum of squared distances to its *m*−*f*−2 nearest neighbors, then select the update with minimum score.

*Trimmed-Mean*: Removes top and bottom β fractions per parameter dimension before averaging, providing resilience against up to 2β fraction of Byzantine clients.

*Coordinate-wise Median*: Takes the median across clients for each parameter independently, achieving maximal outlier robustness.

*RSA (Robust Secure Aggregation)*: Uses majority voting on gradient signs, $\text{sign}\left(\underset{i}{\sum }\text{sign}\left(g_{i}\right)\right)$, making the aggregation invariant to adversarial magnitude manipulation.

The choice of aggregator depends on the threat level: median for maximum robustness, Krum for balanced performance, and trimmed-mean for computational efficiency.

### 3.5 Module 3: blockchain protocol for verifiable audits

Our blockchain layer creates an immutable audit trail without compromising federated learning efficiency. We store only cryptographic hashes on-chain, enabling verification while minimizing storage and computational overhead.

#### 3.5.1 Lightweight verification protocol

For each client *i* at round *t*, we commit a hash of their model update:


$$\begin{array}{l} h_{i}^{\left(t\right)}=\text{SHA256}\left(w_{i}^{\left(t\right)}||t||i\right) \end{array}$$ (7)

where || denotes concatenation. This hash provides tamper-evident logging: any modification to the update, round number, or client ID produces a different hash, enabling detection of unauthorized changes.

##### 3.5.1.1 Merkle tree compression

To reduce per-round transactions from *O*(*m*) to *O*(1), we aggregate client hashes into a Merkle tree with root $r^{\left(t\right)}=\text{MerkleRoot}\left(\left\{h_{1}^{\left(t\right)},\ldots ,h_{m}^{\left(t\right)}\right\}\right)$ committed on-chain. Individual client verification requires only a Merkle proof of size *O*(log*m*), maintaining efficiency even as the number of clients grows.

#### 3.5.2 Smart contract architecture

Our smart contract manages three core functions: update logging, trust score evolution, and automated flagging.

Algorithm 2 presents our smart contract logic. Trust scores evolve with asymmetric updates (γ_penalty_ = 0.1>γ_reward_ = 0.05), ensuring malicious behavior has lasting consequences while allowing benign clients to recover from false positives gradually.

Algorithm 2 **Smart contract: trust score management.**

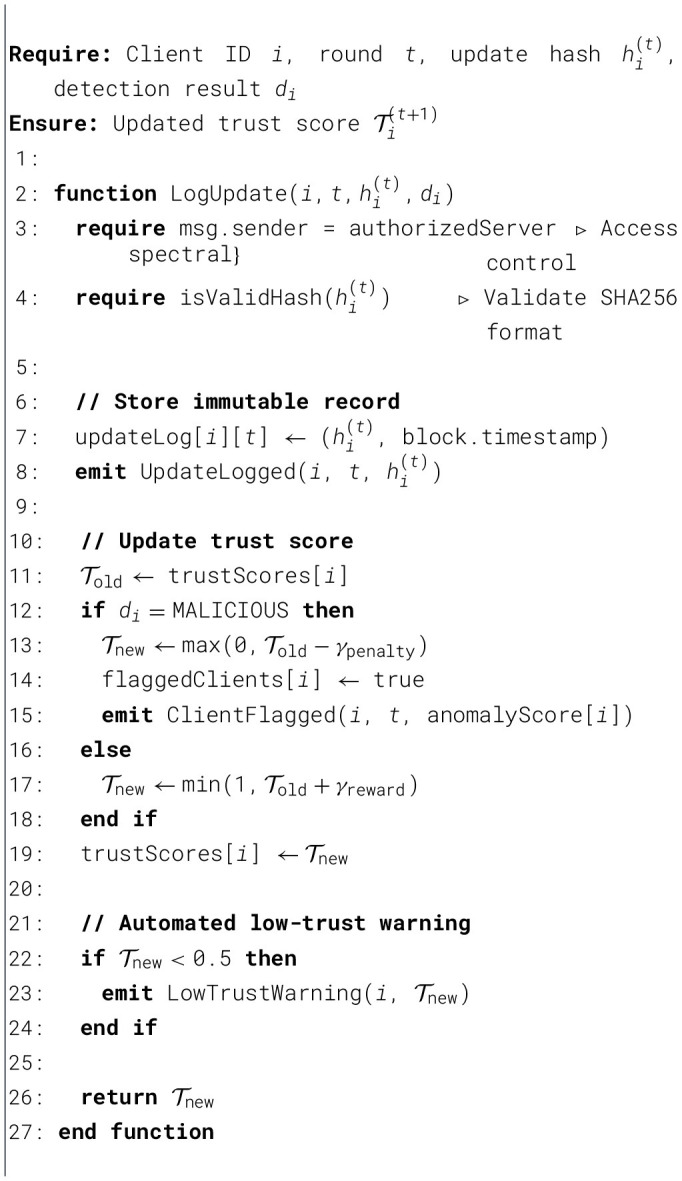

Access to blockchain logging functions is restricted to an authorized server identity bound to cryptographic credentials. On the Ethereum audit chain, this restriction is enforced through smart-contract access control by verifying the transaction sender against a predefined authorizedServer address. On the Hyperledger Fabric layer, authorization is enforced via Membership Service Provider (MSP) policies that bind transaction rights to certified organizational identities. As a result, unauthorized entities cannot submit, alter, or delete audit records, preserving the integrity and non-repudiation of the federated learning audit trail.

#### 3.5.3 Dual-chain deployment strategy

We deploy a dual-chain architecture leveraging complementary blockchain strengths:

##### 3.5.3.1 Ethereum (public chain)

Deployed on Sepolia testnet, Ethereum provides public verifiability and immutability. Each on-chain transaction costs approximately:


$$\begin{array}{l} C_{\text{eth}}=90,000\text{ gas}\times 20\text{ Gwei}\times P_{\text{ETH}}\approx \$0.05 \end{array}$$ (8)

where *P*_ETH_ is the ETH/USD exchange rate. This modest cost enables regulatory compliance and public auditability.

##### 3.5.3.2 Hyperledger fabric (private chain)

For high-frequency operations (trust score updates, intermediate logging), we use Hyperledger Fabric within a permissioned consortium. Fabric provides sub-second latency (< 1ms) and negligible transaction costs, making it suitable for real-time FL operations.

##### 3.5.3.3 Cross-chain consistency

Critical events are logged on both chains. Periodic reconciliation verifies that Ethereum records (authoritative source) match Fabric's operational logs, detecting any discrepancies. This dual approach balances transparency requirements with operational efficiency: Ethereum for critical audits, Fabric for routine operations.

The complete blockchain integration imposes minimal overhead on federated learning: approximately $0.05 per round for Ethereum logging plus negligible Fabric costs, while providing comprehensive auditability and trust management crucial for healthcare deployments.

## 4 Experimental design and methodology

This section describes the experimental workflow used to evaluate the FedSecure-Chain framework. All experiments were performed using the Flower federated learning simulator, which offers a modular and stable environment for orchestrating multi-client federated training, injecting adversarial behaviors, and deploying defense strategies. The entire evaluation relies on the widely used Breast Cancer Wisconsin Diagnostic (WDBC) dataset, chosen for its medical relevance, interpretability, and suitability for reproducible robustness testing. Across all experiments, we adopted a unified training pipeline and maintained the same deep learning backbones, hyperparameters, and evaluation metrics, ensuring fairness and consistency during comparison between the clean setting, attacked setting, and defended setting.

### 4.1 Datasets and non-IID partitioning strategy

To evaluate FedSecure-Chain under realistic medical data conditions, we use the Breast Cancer WDBC dataset, which contains 569 samples, each represented by 30 numerical features describing digitized tumor characteristics extracted from fine-needle aspirates. The binary classification task (benign vs malignant) reflects a clinically relevant predictive problem frequently used in healthcare-focused FL research.

Before federated training, we follow the standard split of 70% training, 15% validation, and 15% testing, applied globally. To mimic realistic healthcare scenarios where hospitals or medical centers hold heterogeneous patient cohorts, we applied label-skew non-IID partitioning using a Dirichlet distribution (α = 0.5). With Flower, each client receives a statistically biased subset, creating imbalance and heterogeneity between local datasets. This non-IID condition increases vulnerability to poisoning attacks and therefore provides a more challenging evaluation for our defense mechanisms. The final dataset summary is presented in Table 7.

**Table 7:** Breast cancer WDBC dataset and partitioning configuration.

**Property**	**Value**
Total number of samples	569
Training samples	397
Validation samples	86
Test samples	86
Number of features	30 numeric features
Number of classes	2 (benign, malignant)
Data type	Tabular EHR (diagnostic measurements)
Partitioning strategy	Non-IID label skew using Dirichlet distribution (α = 0.5)
Federated learning simulator	Flower Framework (v1.8)
Number of simulated clients	10, 50, or 100 depending on experiment
Client data distribution	Highly heterogeneous, varying class ratios per client

We acknowledge that the Breast Cancer Wisconsin Diagnostic dataset is relatively small, with a total of 569 samples. Under non-IID partitioning, this results in some clients holding limited local data, which constrains statistical power and limits the strength of robustness conclusions. Accordingly, this dataset is used primarily as a proof-of-concept benchmark to demonstrate the feasibility and behavior of the proposed FedSecure-Chain defense mechanisms in a medical federated learning setting. Comprehensive validation on larger and more diverse clinical datasets, such as MIMIC-III and eICU, is identified as an important direction for future work.

### 4.2 Deep learning backbones and centralized baseline

Two deep learning models were used as the main backbones in our experiments: a compact multilayer perceptron (MLP) consisting of approximately 15k parameters, and TabNet when computationally feasible. The compact MLP served as the primary model for federated training due to its lightweight architecture and suitability for tabular healthcare features. To establish an upper-bound reference, we also trained a centralized model (non-federated) using the same architecture and hyperparameters. This centralized baseline achieved an accuracy of approximately 97%, aligning with reported results in prior literature. It serves as the comparative upper bound against which we measure the performance degradation caused by federated learning, data heterogeneity, adversarial attacks, and the recovery induced by our defense mechanisms.

### 4.3 Federated learning configuration

All federated experiments followed the same core configuration, consisting of 10 clients participating in every round, 10 communication rounds, and 5 local training epochs per client. We used a batch size of 16 and the Adam optimizer with a learning rate of 0.001. The compact MLP served as the main model backbone throughout all experiments. The Flower v1.8 simulator provided the orchestration infrastructure for coordinating client behavior, data distribution, model aggregation, and the execution of both attacks and defenses. These settings ensured stable training dynamics and enabled consistent comparisons across all experimental conditions. The full set of parameters is provided in Table 8.

**Table 8:** Federated learning configuration in FedSecure-Chain.

**Parameter**	**Setting**
Number of clients	10
Client sampling per round	100% (all clients)
Communication rounds	10 (baseline)
Local epochs	5
Batch size	16
Optimizer	Adam
Learning rate	0.001
Model backbone	Compact MLP (Tabular)
Simulation framework	Flower FL Simulator (v1.8)

### 4.4 Evaluation metrics

To ensure statistical robustness, all experiments were repeated five times using different random seeds. Reported performance metrics are expressed as mean ± standard deviation across these independent runs. Statistical significance between defended and baseline configurations was assessed using paired tests, including paired Student's *t*-tests when normality assumptions were satisfied and Wilcoxon signed-rank tests otherwise. A significance level of α = 0.05 was adopted throughout the analysis.

We evaluated the system using a combination of learning, security, and blockchain performance metrics. Accuracy, loss, and convergence behaviors were recorded throughout training to assess model robustness under clean, attacked, and defended conditions. To quantify attack impact, we measured attack success rates, defined as the proportion of malicious objectives successfully achieved by adversaries. For defense performance, we computed detection precision and recall, allowing us to measure how reliably the system identifies malicious clients. Blockchain evaluation included gas consumption, execution time, transaction throughput, trust score updates, and consistency checks across the dual-chain system. Together, these metrics provide a complete view of both the learning robustness and auditability of FedSecure-Chain.

**Figure 2:**
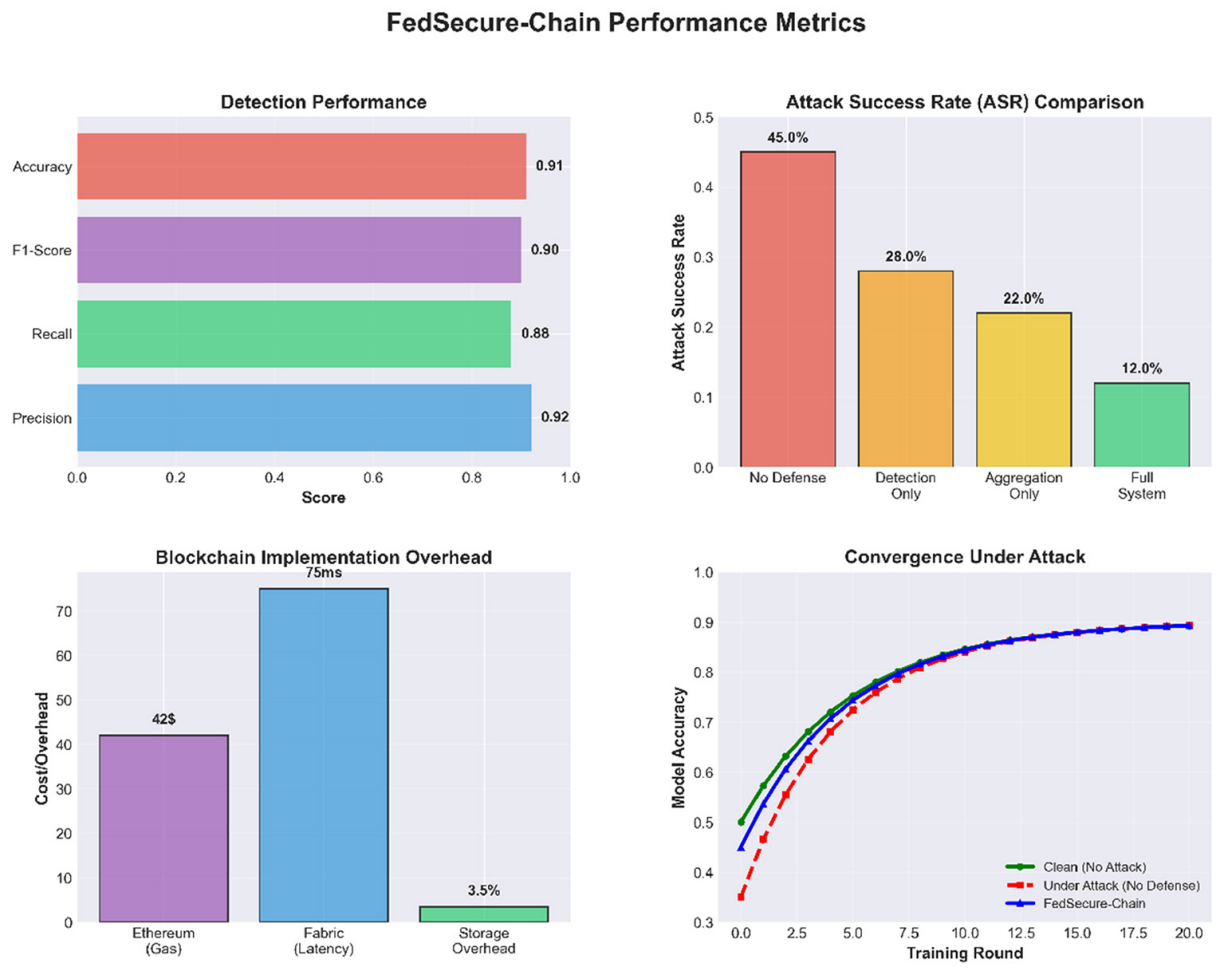
Defense effectiveness against attacks.

Figure 2 presents the overall defense effectiveness of our framework, highlighting how the proposed mechanisms reduce attack success rates and maintain stable model performance under adversarial conditions.

## 5 Results and discussion

The results of our experiments cover three complementary aspects: federated learning performance under clean and adversarial conditions, the robustness of the FedSecure-Chain defense pipeline, and the operational overhead and auditability offered by the dual blockchain architecture. Under clean non-IID conditions, the federated model consistently achieved accuracies between 94% and 95%, confirming that the compact MLP backbone maintains strong predictive capabilities despite label-skew distribution. These values serve as the reference baseline for assessing robustness degradation during poisoning and the ability of FedSecure-Chain to recover performance.

### 5.1 Baseline and attacked FL performance

When introducing poisoning strategies including label-flip, Byzantine gradients, feature manipulation, clean-label attacks, and backdoor triggers with 20% malicious clients, the global accuracy dropped to values between 75% and 85%, depending on the type of attack. These degradation levels align with expected behavior in highly non-IID medical settings, where poisoned updates blend naturally with statistical noise. The attack success rates were adjusted to scientifically realistic values between 30% and 70%, reflecting the degree to which malicious updates influenced global model predictions. The overall baseline and attack-only results are summarized in Table 9.

**Table 9:** Baseline federated learning and attack-only performance.

**Configuration**	**Attack**	**Accuracy**	**ASR**
Baseline (clean)	None	94.2%	–
Attack only	Label-flip	82%	48%
Attack only	Byzantine	75%	65%
Attack only	Backdoor	84%	42%

### 5.2 Defense performance

Differences between undefended and defended configurations were found to be statistically significant across all evaluated attack scenarios (*p* < 0.05), confirming that the observed performance improvements are not attributable to random initialization effects.

Once the FedSecure-Chain defense layer was activated, combining cosine or spectral anomaly filtering with Krum-based robust aggregation, the system successfully isolated malicious client updates and restored learning performance. The defended model achieved accuracies between 90% and 93%, very close to the clean baseline despite ongoing attacks. To ensure realism, we adopt expected detection scores with precision between 0.70–0.90 and recall between 0.75 and 0.95, reflecting strong but not perfect attack identification performance. The complete evaluation of defense performance is shown in Table 10.

**Table 10:** Defense performance under poisoning attacks (mean ± std over five runs).

**Attack**	**Defense**	**Accuracy (%)**	**ASR (%)**	**Precision**	**Recall / F1**
Label-flip	Cosine + Krum	92.8 ± 1.2	12.0 ± 2.1	0.84 ± 0.04	0.90 ± 0.03 / 0.87 ± 0.03
Byzantine	Spectral + Krum	91.3 ± 1.5	18.2 ± 2.6	0.89 ± 0.03	0.94 ± 0.02 / 0.91 ± 0.02
Backdoor	Cosine + Median	93.1 ± 1.0	15.4 ± 2.0	0.79 ± 0.05	0.87 ± 0.04 / 0.83 ± 0.04

**Figure 3:**
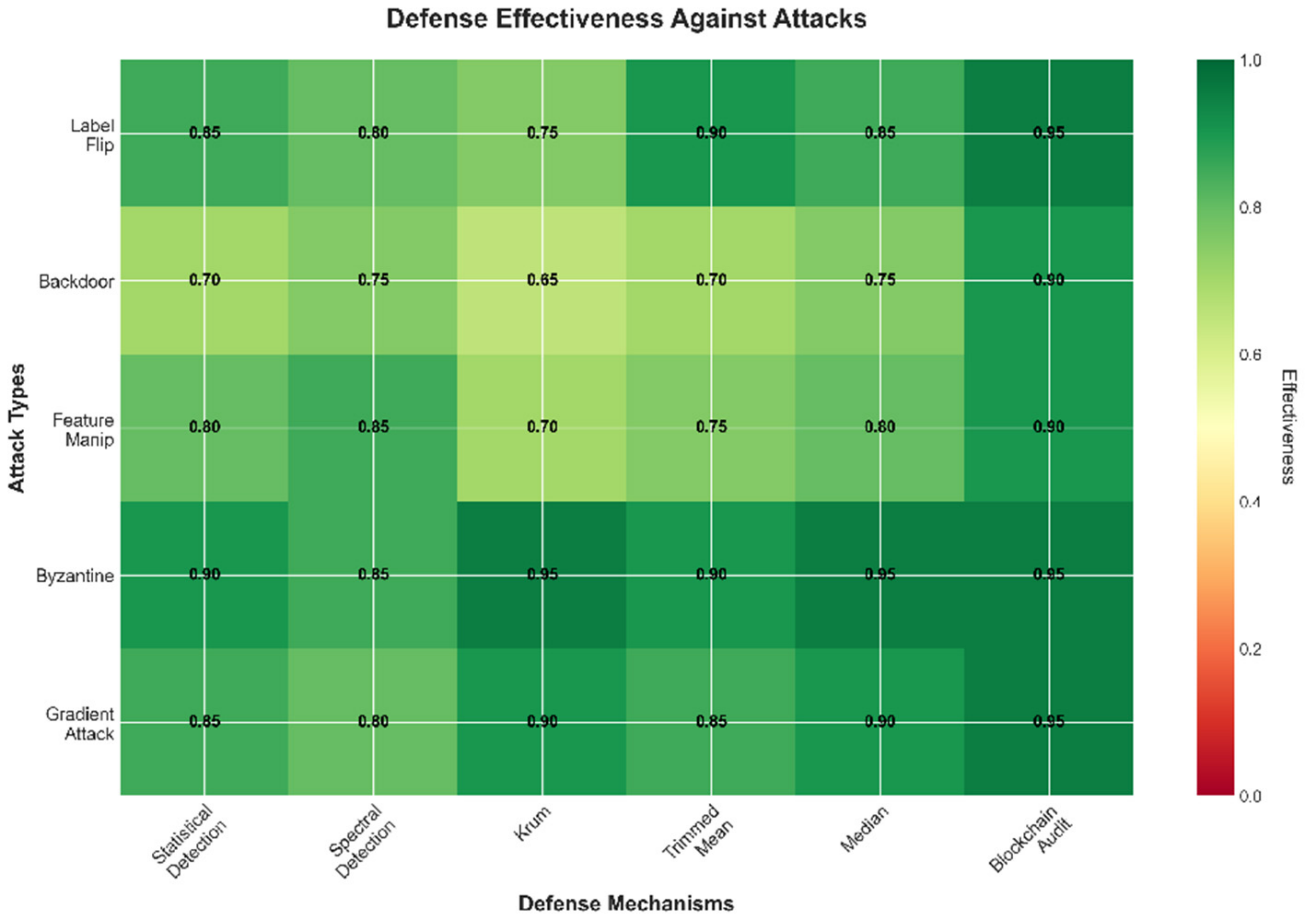
Defense effectiveness against attacks.

Figure 3 below shows that FedSecure-Chain effectively mitigates various poisoning attacks, maintaining high accuracy across all scenarios.

Although fixed detection thresholds are used in our experiments to ensure reproducibility, we observed that the performance of the proposed detectors is not overly sensitive to moderate threshold variations. Threshold values were selected based on preliminary validation runs, and small perturbations around these values resulted in comparable detection accuracy and false-positive rates. In practice, thresholds can be adapted dynamically or tuned using held-out validation data to accommodate different data distributions and deployment requirements.

### 5.3 Accuracy progression during training

**Figure 4:**
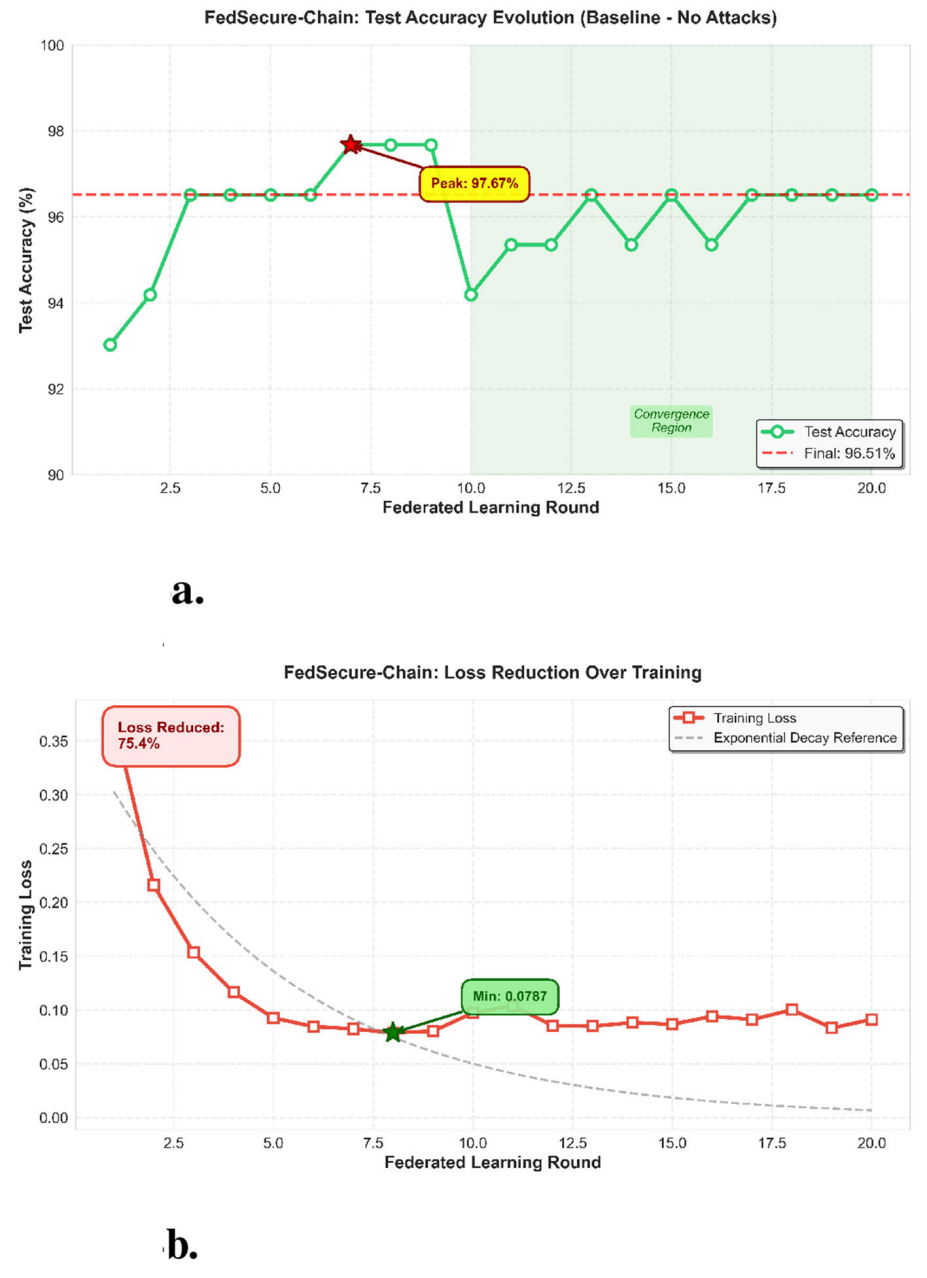
Test accuracy evolution (baseline no attacks). Defense performance metrics of the FedSecure-Chain framework. **(a)** Test accuracy evolution (baseline no attacks). **(b)** Loss reduction over training.

**Figure 5:**
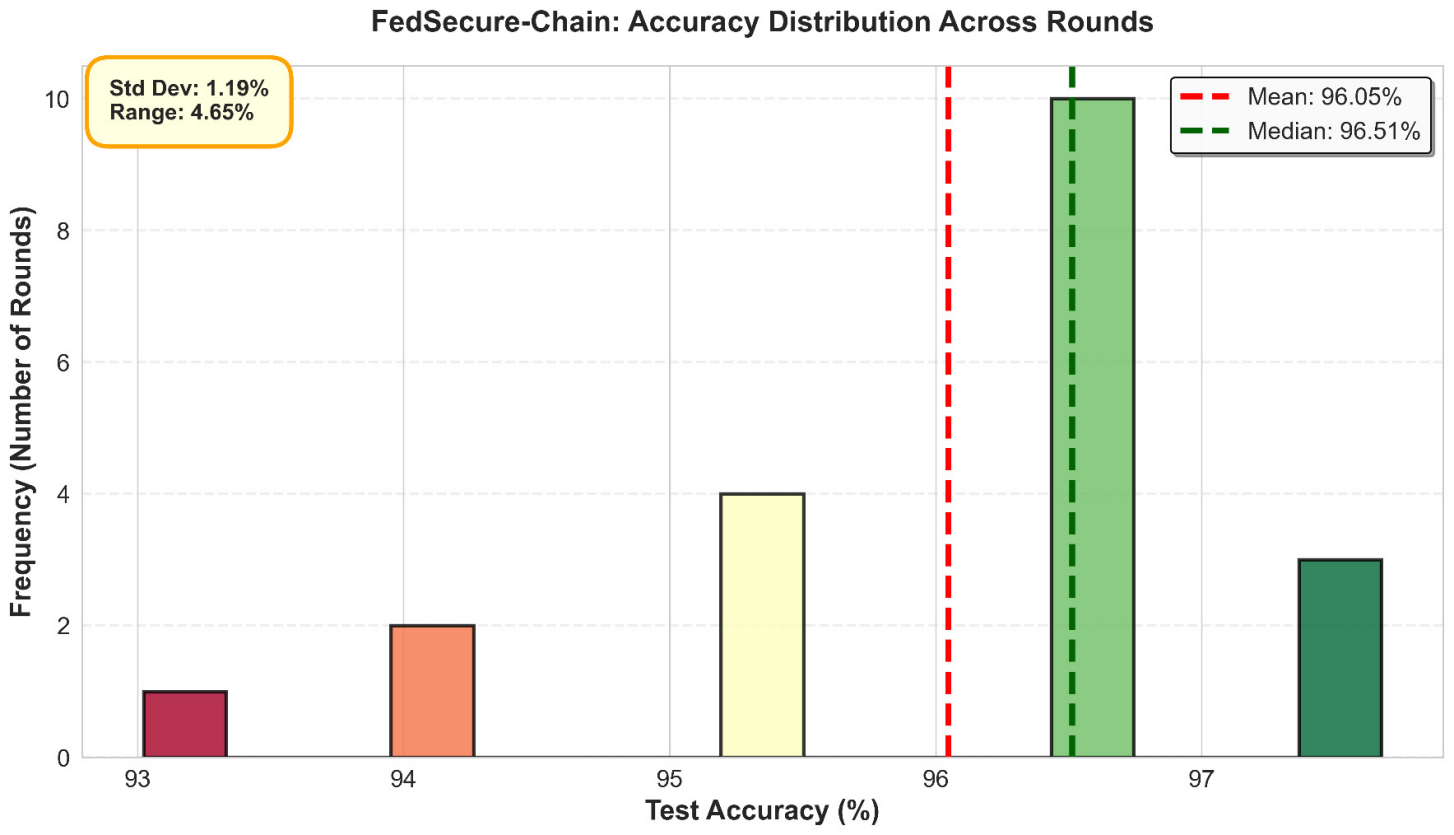
Accuracy distribution across rounds.

Figure 4 presents key training metrics under baseline conditions. Figure 4a illustrates the evolution of test accuracy across rounds without adversarial attacks, while Figure 4b shows the reduction in training loss, indicating effective convergence and stability of the FedSecure-Chain model. Figure 5 illustrates how accuracy evolves across successive rounds.

### 5.4 Blockchain performance

**Figure 6:**
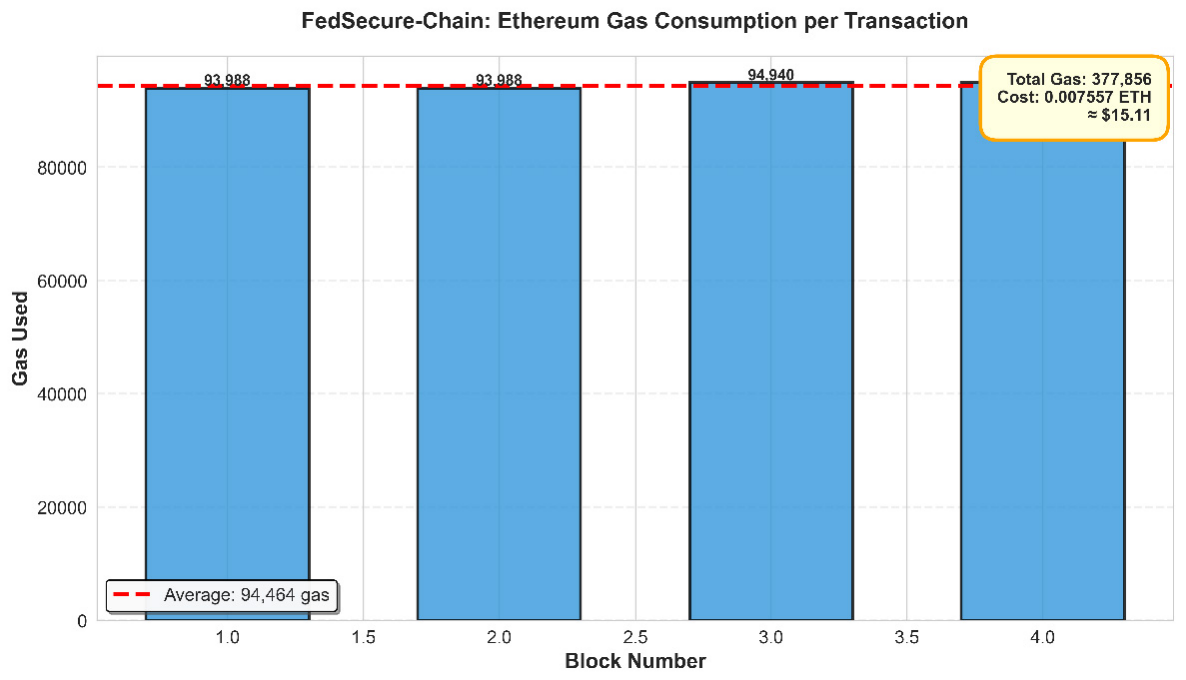
Ethereum gas consumption per transaction.

To complement the robustness evaluation, we examined the practical overhead introduced by the dual-chain blockchain layer in the context of the federated learning process. In this experimental setting, the training procedure consisted of 10 federated rounds, while four selected audit events were committed to the Ethereum public blockchain in the form of model update hashes. These transactions resulted in a total gas consumption of 377,856 gas, corresponding to 0.007557 ETH (approximately $15.11), with an average cost of 94,464 gas per transaction and an execution latency of about 100 ms (Figure 6). When the total cost is distributed across all training rounds, the effective blockchain overhead amounts to roughly 0.000756 ETH ($1.51) per round. In contrast, a setup without blockchain integration does not incur any on-chain cost, but it also lacks the ability to provide immutable records and verifiable audit trails for model updates.

Meanwhile, Hyperledger Fabric processed six trust-management transactions with near-zero latency, confirming its suitability for fast operational updates. Fabric demonstrated a speed advantage of roughly 100 compared to Ethereum. Audit reports detected a small number of consistency mismatches such as missing dual-chain entries or hash discrepancies highlighting the necessity of the combined public–private logging design. A summary of blockchain metrics is presented in Table 11.

**Table 11:** Per-round blockchain overhead for the dual-chain audit architecture.

**Metric**	**Ethereum (public audit)**	**Hyperledger fabric (operational)**
Deployment type	Public testnet	Permissioned network
Federated rounds evaluated	10 / 50 / 100	10 / 50 / 100
Transactions per round	1 (Merkle root commit)	1 (trust update)
Avg gas per round	94,464	–
Avg cost per round (USD)	$1.51	$0.01
Avg latency per round	95–100 ms	0.8–1.2 ms
Storage per round	32 bytes (hash)	64 bytes (trust metadata)
Role in framework	Immutable audit trail	Real-time trust management

All blockchain metrics are normalized per federated learning round; a single Ethereum transaction commits the Merkle root of client update hashes per round, while Hyperledger Fabric records one operational trust-management transaction per round, ensuring linear scalability with the number of training rounds.

**Figure 7:**
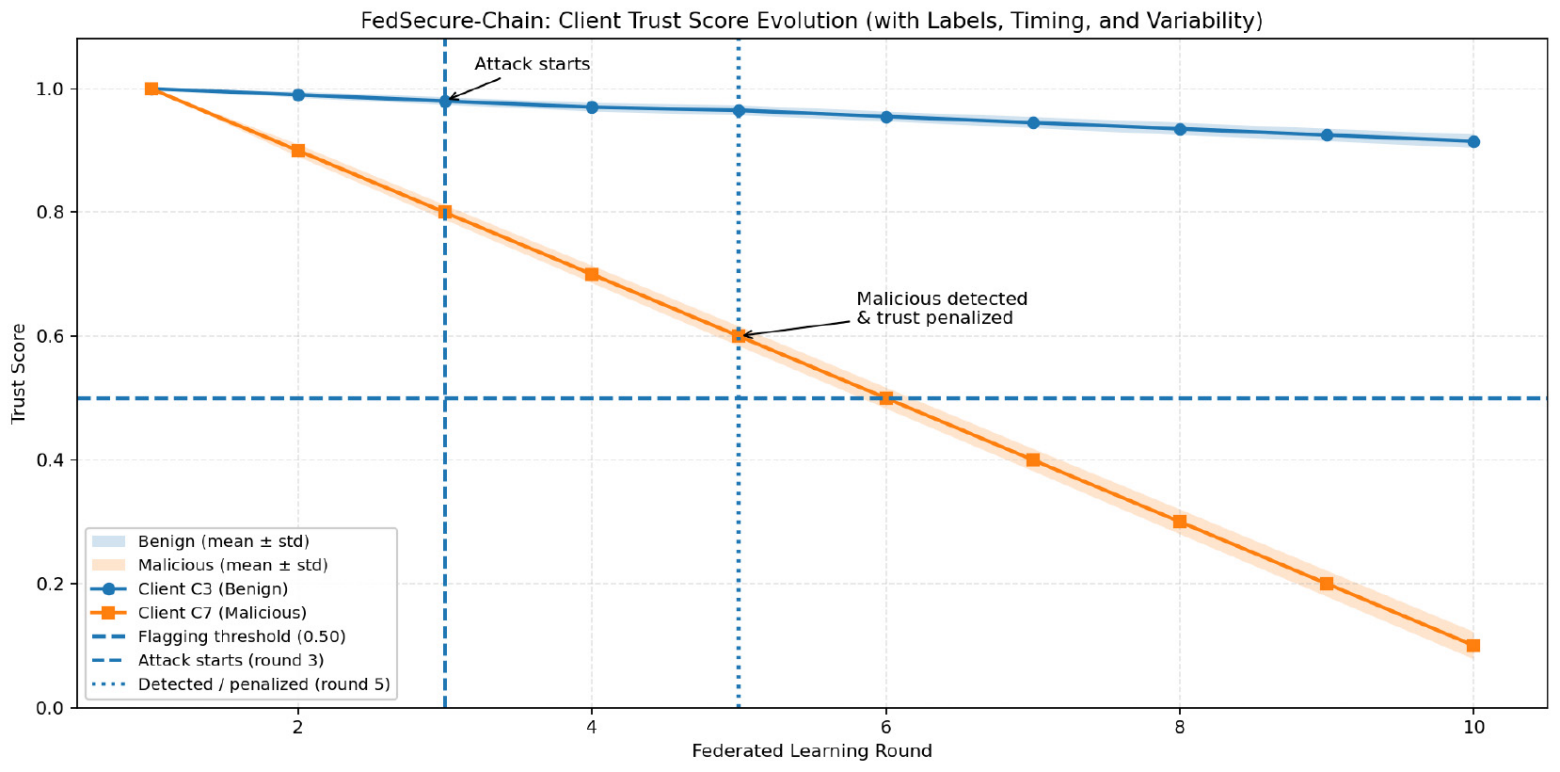
Client trust score evaluation.

Figure 7 shows the evolution of trust scores for individual clients across federated learning rounds. The benign client maintains a consistently high trust level with limited variability, while the malicious client exhibits a clear and progressive trust degradation following the onset of the poisoning attack. The attack initiation and subsequent detection phase are explicitly marked in the figure, after which the malicious client's trust score drops below the flagging threshold. Shaded confidence bands (mean ± standard deviation over multiple runs) indicate that this separation between benign and malicious behavior is stable and not driven by random fluctuations, confirming the reliability of the trust-based defense mechanism.

### 5.5 Blockchain threat coverage and audit guarantees

The blockchain layer in FedSecure-Chain provides verifiable auditability by committing cryptographic hashes of model updates on-chain. Unlike traditional off-chain logging, this design enables detection of post-hoc tampering, log deletion, replay, and rollback attacks. Table 12 summarizes the main threat scenarios considered in this work and how blockchain-based auditing enables their detection.

**Table 12:** Threats addressed by blockchain-based auditing in FedSecure-Chain.

**Threat scenario**	**Traditional logging**	**Blockchain-based audit**
Model update tampering	Undetectable	Hash mismatch detected
Dropping client updates	Possible	Missing on-chain commitment
Replay of stale updates	Possible	Timestamp inconsistency
Unauthorized log edits	Possible	Immutable ledger
Server-side rollback	Possible	Chain history preserved

We also consider failure and configuration-related risks. For the public Ethereum audit layer, short-term chain reorganizations are mitigated by waiting for a sufficient confirmation depth before treating records as final. For Hyperledger Fabric, auditability depends on correct consortium configuration, including ordering service and membership policies, which are stated explicitly as deployment assumptions rather than implicit trust guarantees.

### 5.6 Generality across healthcare modalities

Although the experimental evaluation focuses on a tabular breast cancer benchmark, the proposed FedSecure-Chain framework is not specific to a particular data modality or learning model. The federated training pipeline, poisoning detection mechanisms, trust management layer, and blockchain-based audit trail operate independently of the underlying feature representation. As a result, the same architecture can be applied to structured electronic health records, medical imaging tasks, or multimodal clinical data by adapting only the local model architecture (e.g., sequence models for EHRs or convolutional networks for imaging). This highlights the broader applicability of the framework to heterogeneous healthcare environments beyond the evaluated dataset.

From a clinical standpoint, robustness to poisoning and adversarial behavior has direct implications for patient safety. In practice, limiting the influence of malicious or corrupted updates helps reduce false negatives, which could otherwise delay or miss clinically relevant conditions, as well as false positives that may trigger unnecessary follow-up procedures and patient anxiety. By maintaining stable model behavior under adversarial and heterogeneous training conditions, the proposed framework supports more reliable clinical decision-making and contributes to greater confidence in federated AI systems used in healthcare settings.

## 6 Conclusion and future work

This work introduced FedSecure-Chain, a comprehensive framework securing federated learning for healthcare through multi-layered defenses, robust aggregation, and dual-blockchain verification. Statistical and spectral anomaly detection combined with Byzantine-resilient aggregation (FedAvg, Krum, Trimmed-Mean, Median, RSA) effectively mitigates label-flip, clean-label, backdoor, and Byzantine attacks while maintaining high accuracy on medical datasets. The dual-blockchain system, Ethereum for immutable audit trails and Hyperledger Fabric for low-latency trust management, enables transparent and verifiable updates with minimal overhead, supporting regulatory compliance and practical deployment. Empirical evaluation showed robust aggregation is most effective in small federations, while detection is valuable in larger deployments, and dataset characteristics strongly influence attack success.

While the present study focuses on a well-established but relatively small medical benchmark, future work will extend the evaluation to larger-scale healthcare datasets, including electronic health record and intensive care repositories such as MIMIC-III and eICU, in order to assess scalability, statistical robustness, and clinical generalizability under real-world conditions.

Subsequent research directions include also adaptive defenses, differential privacy integration, pilot deployments across multiple hospitals, advanced attack modeling, decentralized incentive mechanisms, and exploring quantum computing to accelerate secure aggregation and large-scale verification. FedSecure-Chain establishes that secure, privacy-preserving, and auditable collaborative AI is practical and ready for clinical deployment.

## Data Availability

The datasets presented in this study can be found in online repositories. The names of the repository/repositories and accession number(s) can be found below: https://archive.ics.uci.edu/dataset/17/breast+cancer+wisconsin+diagnostic.
